# Long-term effects on luminal and mucosal microbiota and commonly acquired taxa in faecal microbiota transplantation for recurrent *Clostridium difficile* infection

**DOI:** 10.1186/s12916-016-0698-z

**Published:** 2016-10-11

**Authors:** Jonna Jalanka, Eero Mattila, Hanne Jouhten, Jorn Hartman, Willem M. de Vos, Perttu Arkkila, Reetta Satokari

**Affiliations:** 1 Immunobiology Research Program and Department of Bacteriology and Immunology, Faculty of Medicine, University of Helsinki, Helsinki, Finland; 2 Department of Infectious Disease, Helsinki University Central Hospital, Helsinki, Finland; 3 Laboratory of Microbiology, Wageningen University, Wageningen, The Netherlands; 4 Department of Gastroenterology, Helsinki University Central Hospital, Helsinki, Finland; 5 PO Box 21, Haartmaninkatu 3, 00290 Helsinki, Finland

**Keywords:** Bacteriotherapy, Microbiome, Universal donor, Mucosal bacteria

## Abstract

**Background:**

Faecal microbiota transplantation (FMT) is an effective treatment for recurrent *Clostridium difficile* infection (rCDI). It restores the disrupted intestinal microbiota and subsequently suppresses *C. difficile*. The long-term stability of the intestinal microbiota and the recovery of mucosal microbiota, both of which have not been previously studied, are assessed herein. Further, the specific bacteria behind the treatment efficacy are also investigated.

**Methods:**

We performed a high-throughput microbiota profiling using a phylogenetic microarray analysis of 131 faecal and mucosal samples from 14 rCDI patients pre- and post-FMT during a 1-year follow-up and 23 samples from the three universal donors over the same period.

**Results:**

The FMT treatment was successful in all patients. FMT reverted the patients’ bacterial community to become dominated by Clostridium clusters IV and XIVa, the major anaerobic bacterial groups of the healthy gut. In the mucosa, the amount of facultative anaerobes decreased, whereas Bacteroidetes increased. Post-FMT, the patients’ microbiota profiles were more similar to their own donors than what is generally observed for unrelated subjects and this striking similarity was retained throughout the 1-year follow-up. Furthermore, the universal donor approach allowed us to identify bacteria commonly established in all CDI patients and revealed a commonly acquired core microbiota consisting of 24 bacterial taxa.

**Conclusions:**

FMT induces profound microbiota changes, therefore explaining the high clinical efficacy for rCDI. The identification of commonly acquired bacteria could lead to effective bacteriotherapeutic formulations. FMT can affect microbiota in the long-term and offers a means to modify it relatively permanently for the treatment of microbiota-associated diseases.

**Electronic supplementary material:**

The online version of this article (doi:10.1186/s12916-016-0698-z) contains supplementary material, which is available to authorized users.

## Background

The incidence of *Clostridium difficile* infections (CDI) has increased, with up to 50 % of patients developing recurrent infections [1, 2]. The bacterium is the main etiological agent of antibiotic-associated diarrhoea, causing a major burden to the healthcare system [3–5]. Diverse intestinal microbiota provides colonisation resistance against pathogens and perturbations to the normal microbiota introduced by an antibiotic treatment is a key step in CDI pathogenesis [5]. Traditionally, CDI is treated with metronidazole or vancomycin and, more recently, with fidaxomicin and rifaximin [2, 6, 7]. These antimicrobials devastate the intestinal microbiota even further. If *C. difficile* spores persist after antibiotic treatment they can germinate and proliferate in the absence of suppressing microbiota and, as a consequence, the patient may enter a vicious cycle of recurrent CDI (rCDI) infections [8]. Further, the emerging antibiotic-resistant variants of *C. difficile* call for alternative treatment options [9].

Faecal microbiota transplantation (FMT) is highly effective in treating rCDI [10–13]. FMT from a healthy, pre-screened donor is placed into the patient’s duodenum, cecum or rectum where it restores the diversity and composition of the disrupted microbiota and subsequently suppresses *C. difficile* [9, 11–18]. Emerging evidence suggests that FMT also restores secondary bile acid metabolism, which is impaired in rCDI and possibly has a role in disease development [19, 20]. Several studies have followed the short-term stability of the transplanted microbiota and constituted that, overall, FMT-induced changes tend to persist over time [14, 16, 17]. Further, FMT’s long-term clinical efficacy and safety have been demonstrated [10, 11, 21]. However, the long-term effects of FMT on microbiota have not been previously addressed, with prior work focusing on the effects on faecal microbiota rather than on the distinct ecosystem of mucosa.

Understanding the mechanistic basis of FMT treatment and the minimum microbial components necessary for a successful outcome are vital. Preliminary studies have been conducted, with evidence from a rCDI mouse model suggesting that a mixture of intestinal bacteria could be used instead of faecal material [22]. More recently, bacterial mixtures comprising over 30 strains were shown to resolve rCDI in two patients [23]. These results suggest that an effective treatment of CDI based on defined mixtures of bacteria might be feasible in the near future.

In this study, we aimed to build on the existing knowledge by concentrating on the long-term effects of FMT on the faecal microbiota as well as characterising rectal mucosal microbiota pre- and post-treatment. We used a universal donor approach, where several patients received their transplant from the same donor. This facilitated a controlled analysis of FMT-induced microbiota changes and the identification of key bacterial taxa that are commonly established in the gut of CDI patients. Thereby, we aimed to investigate the possibility of a commonly acquired core microbiota underlying the efficacy of the FMT treatment and which could be used as a basis for the design of bacteriotherapeutic formulations.

## Methods

### Patients

**Fig. 1:**
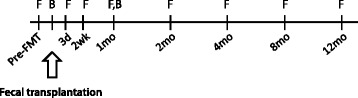
Study design. Four to eight faecal samples were collected from 14 patients and three donors over the 1-year study period, in addition to two biopsy samples (from 10 patients only). *F* faecal sample, *B* biopsy sample

The intestinal microbiota of 14 rCDI patients treated with FMT was analysed (Table 1; see the Additional file 1: Table S1 for detailed patient information and Fig. 1 and Additional file 1: Figure S1 for sample collection). All patients had laboratory-confirmed rCDI despite antimicrobial treatment and were refractive to standard therapy. One patient, P13, received FMT after one relapse only. She had previously suffered from three CDIs during the past 3 years, always coinciding with antibiotic treatment for other indications. The latest *C. difficile* infection started after a course of doxycycline and, due to the patient’s history, FMT was considered as a suitable treatment and the patient was included in the study. The previously described clinical inclusion and exclusion criteria were followed [21]. The study was approved by the Ethics Committee of Hospital District of Helsinki and Uusimaa Finland (DnroHUS124/13/03/01/11). Patients were informed about the possible risks of FMT and they all provided informed consent.

**Table 1:** Patient demographics

Patient no.	Age, years	Sex	Donor	Faecal material	Outcome
P1	63	Male	D2	Frozen	Resolution
P2	45	Male	D2	Frozen	Resolution
P3	88	Female	D2	Frozen	Resolution
P4	82	Female	D2	Frozen	Resolution
P5	81	Female	D2	Frozen	Resolution
P6	58	Female	D3	Fresh	Resolution
P7	67	Male	D1	Fresh	Resolution
P8	31	Female	D3	Frozen	Resolution
P9	35	Female	D3	Fresh	Resolution
P10	81	Male	D3	Fresh	Resolution
P11	80	Female	D3	Frozen	Resolution
P12	20	Female	D3	Frozen	Resolution
P13	57	Female	D2	Frozen	Resolution
P14	44	Female	D3	Frozen	Resolution

### Donors and preparation of faecal transplants

Three healthy Finnish females, aged 35–42 years and with a normal body mass index (average 23.5, SD = 2.1) acted as universal faecal donors (D1–D3) and provided follow-up faecal samples (Fig. 1). The donors were screened as described previously [11]. In short, they did not have any gastrointestinal symptoms, had not taken antibiotics for the past 6 months, and were negative in *C. difficile* culture and toxin A/B test. They were also negative for growth on selective culture for enteric bacterial pathogens and light microscopy on ova and parasites from faeces and as well tests for HBV, HCV, HIV-1, HIV-2 and *Treponema pallidum* from serum. Further tests included total blood count, C-reactive protein, creatinine and liver enzyme levels from blood.

The preparation of faecal suspensions for immediate use and for freeze-storing at –80 °C was performed as described recently by using 30 g of faecal material [21]. The patients received an infusion of either fresh faeces or previously frozen sample (Table 1). The frozen donor samples were stored at −80 °C for a maximum of 4 months prior to transplantation.

### Faecal microbiota transplantation

The patients were treated with vancomycin pre-FMT and the medication was discontinued in average 36 hours prior to treatment. Patients cleansed their bowels before FMT with polyethylene glycol [11, 21]. The faecal suspension was infused into the cecum. Patients were advised to contact the hospital if they had diarrhoea or other symptoms after FMT. Persisting diarrhoea with a positive *C. difficile* toxin stool test was considered as a treatment failure. The patients came for the second biopsy 1 month after the FMT (bowel not cleansed). In addition, the patients were paid a home visit 2 months after the transplantation and twice more during the 1-year follow-up period to collect the stored faecal samples, which were kept in their home freezers at −20 °C for 4 months.

### Samples and DNA extraction

The baseline faecal samples were taken before the colonoscopy at home by the patients and brought to the clinic. The follow-up samples were frozen at −20 °C immediately after defecation and stored in the patients home freezers for maximally 4 months until transfer to the laboratory for further analysis. Rectal biopsies were taken from the patients during the FMT (B0) and at 1 month post-FMT (B1) by proctoscopy (bowel not cleaned) and stored at −80 °C until further processing. The patients and donors collected the baseline (F0) and follow-up (F1–F7) faecal samples (Fig. 1). Microbial DNA from patients (n = 131) and donors (n = 23) was extracted as described previously for biopsies and faecal samples according to current standard operation procedures, including a mechanical disruption of bacterial cells [24–26].

### Microbiota analysis

Microbiota analysis was conducted with a benchmarked and validated phylogenetic microarray [27–30]. It covers the V1 and V6 hypervariable regions of the 16S rRNA gene and targets over 1000 bacterial taxa detected in the human GI tract covering the major species. The raw signal intensities were normalised as described previously [29]. For faecal samples, the technical replicates with correlation over 0.96 were accepted for further analysis, and for biopsies and pre-FMT samples a slightly lower quality (over 0.95) was accepted due to the low microbial diversity in the samples. Pre-FMT samples from P13 did not meet these quality standards and were excluded from the analysis. The raw signal intensities were normalised as described previously and min-max algorithms were used for the between sample normalisation [29]. The probe-signal intensities were summarised to 130 genus-like and 22 phylum-like taxonomic groups.

The adherence of bacteria from donor faeces to 7-day-old Caco-2 cells was conducted as described previously [31] and detailed in Additional file 1. The amounts of adhered bacteria were analyzed with MiSeq sequencing of the 16S rRNA gene (detailed in Additional file 1).

### Statistical analysis

All data analyses were carried out with logarithm-transformed data and performed using R (version 3.1.1). The similarity of the microbiota was determined using Spearman’s rank correlation (ρ). In the analysis comparing the similarity of patients’ microbiota and their own donors, subject P13 was excluded due to both Crohn’s disease and multiple antibiotic treatments during the follow-up period. Microbial diversity, a measure of microbial richness and evenness, was calculated by using the inverse Shannon diversity index. The variation in the data was visualised with principle component analysis (PCA). The differences between time points, similarity and diversity were tested with analysis of variance (ANOVA) with Tukey’s honest significant differences post hoc analysis. The changes in the individual bacterial taxa between time points were assessed with a linear mixed model. All resulting *P* values were adjusted for multiple comparisons using Benjamini–Hochberg false discovery rate and *P* values below 0.05 were considered significant. The microbial profile separating the pre- and post-FMT groups was identified with redundancy analysis using bootstrap aggregation (baggedRDA) as described previously [32]. In determining the therapeutic core, a detection threshold of < 2.9 log10 intensity, corresponding to approximately 0.13 % relative abundance from the total bacterial amount, was used here.

## Results

### FMT resolved rCDI and restored healthy microbiota profiles in patients

The FMT treatment cleared rCDI from all patients. A single individual (P3) mistakenly restarted vancomycin after transplantation and developed CDI. She was treated successfully with a second FMT and remained asymptomatic throughout the follow-up period (for detailed analysis see Additional file 1: Figure S2).

**Fig. 2:**
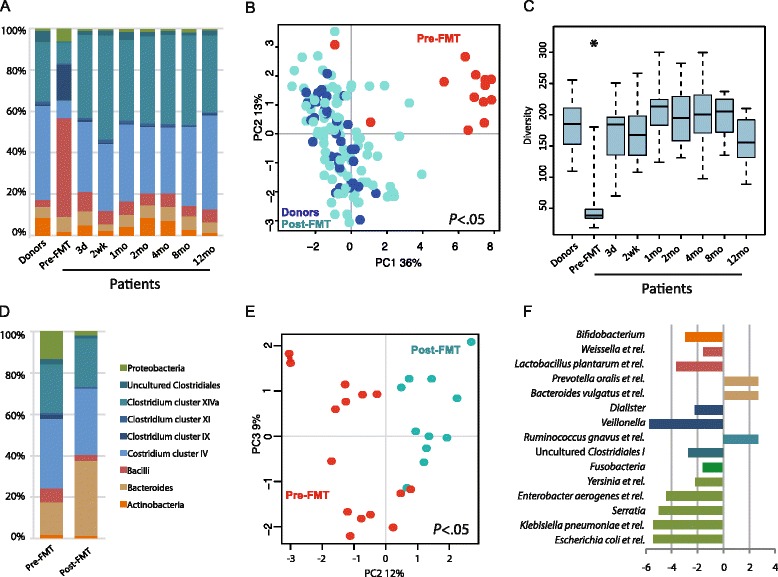
Donors’ microbiota and alterations in the patients’ faecal and mucosal microbiota before and after faecal microbiota transplantation (FMT) treatment. **a** The average microbial composition in faecal samples (see panel d for bacterial groups). Donors’ microbiota shown as average from all time points. **b** Principle component analysis (PCA) from genus level bacterial groups in faecal samples; donors’ samples in dark blue, patients’ pre-FMT samples coloured red and post-FMT samples coloured turquoise. **c** Microbial diversity in faecal samples measured from patients and donors (average from all time points), statistical significance from other time points indicated with an asterisk. **d** The average microbial composition in patients’ mucosal samples. **e** PCA from genus level bacterial groups in patients’ mucosal samples, pre-FMT samples coloured red (patients with one sample n = 13 and patient P3 with 2 samples, see Additional file 1: Table S1) and post-FMT samples (patient n = 11) coloured turquoise. **f** The fold change of genus level bacterial groups was significantly different in the pre- and post-FMT mucosal samples. **d** Phylum level taxonomy

The donors’ microbiota was typical for healthy adults [28, 33–35], dominated by Firmicutes (85.0 %), Actinobacteria (8.5 %) and Bacteroidetes (5.3 %) (Fig. 2a) and showed significant individual-specific profiles. In contrast to the healthy donors, the patients’ microbiota pre-FMT was extremely different. At the highest taxonomical level, 14 out of the 23 detected phylum-like taxa differed significantly between the donors and pre-FMT patients (*P* < 0.05, Fig. 2a). The low levels of Clostridia and high levels of Bacilli and Proteobacteria contributed to the majority of the detected differences. When determining the genus-like taxa separating the pre-FMT patients and donors, we found 69 taxa to be significantly differently abundant in these two groups (Additional file 1: Table S2). Among them, there were 15 genus-like taxa that were increased in abundance by over 10-fold, including bacteria related to *Coprococcus eutactus* (fold change (FC) = 24.71, *P* < 0.05), *Ruminococcus obeum* (FC = 23.05, *P* < 0.05) and *Subdoligranulum variable* (FC = 22.21, *P* < 0.05). Additionally, two genus-level taxa decreased drastically in abundance after FMT, i.e. bacteria related to *Lactobacillus plantarum* (FC = −24.18, *P* < 0.05) and *Veillonella* (FC = −40.64, *P* < 0.05).

There was a dramatic difference in both diversity and microbiota composition after the FMT-treatment. The patients’ microbial diversity increased significantly as early as 3 days post-FMT to resemble the donors and remained in this range for up to 1 year (Fig. 2c). A similar trend was observed with the microbial composition, where patients’ microbiota post-FMT resembled that of the donors throughout the follow-up period (Fig. 2a). This compositional shift is also seen from the unsupervised PCA plot, where 36 % of the microbial variation was introduced by the treatment (Fig. 2b). There was no significant difference between any of the follow-up samples or healthy controls in the PCA.

### FMT-induced microbiota changes in the mucosal surface

Microbiota changes in the intestinal mucosa of CDI patients have not been previously addressed. Interestingly, the changes of microbial profiles from rectal biopsies before and after FMT-treatment were different than what was observed in the faecal samples. The pre-treatment mucosal sample was significantly enriched with members of Clostridium cluster IX, Proteobacteria, Bacilli and uncultured Clostridiales, which were reduced post-FMT, whereas Bacteroidetes were increased after treatment (all, *P* < 0.05, Fig. 2d). Furthermore, when analysing the effect of FMT on the genus-level taxa, there was a significant difference between pre- and post-treatment groups, which were separated in PCA (*P* < 0.05, Fig. 2e). The separation was caused by 15 genus-level taxa (Fig. 2f). The largest difference was introduced by the 2.7-fold increase of the members of Bacteroidetes phylum, including *Bacteroides vulgatus-* and *Prevotella oralis*-related taxa after FMT, whereas members of the Proteobacteria phylum were decreased on average by 4.6-fold and bacteria related to Clostridium cluster IX, such as *Veillonella* spp., decreased. A baggedRDA analysis further supported the observed differences in the mucosal microbiota before and after FMT and confirmed that Proteobacterial and Clostridial species are decreased and the Bacteroidetes species are enriched after the FMT (Additional file 1: Figure S3). Surprisingly, FMT did not increase microbial diversity in the mucosa (Additional file 1: Table S3).

To analyse the adherence of donor faecal bacteria to intestinal epithelium in vitro we studied the most frequently used donor D3 and allowed the faecal sample to bind to the Caco-2 cell culture. The attached bacteria were analysed using 16S rRNA sequencing. Previously, high-throughput sequencing and the microarray platform used in this study have been shown to produce comparable data, particularly at high taxonomic level [30] and, therefore, we considered that it is adequate to analyse the Caco-2 adherent phyla with MiSeq sequencing. Interestingly, the in vitro result replicated our in vivo findings of increased levels of Bacteroidetes in the mucosa by showing a drastic decrease of the Firmicutes/Bacteroidetes ratio from 31.31 to 7.45 in the faecal and Caco-2 adhered samples, respectively. This further suggests that specific bacteria from the faecal material are selected to the mucosal compartment.

### Donor-specific microbiota established in the patients and retained for up to 1 year post-FMT

**Fig. 3:**
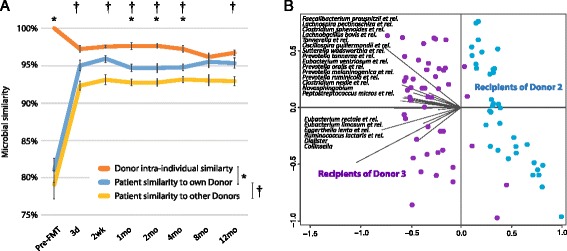
Microbiota stability and donor specific microbiota signatures. **a** Similarity of the patients microbiota to their own donors’ microbiota is significantly higher than the similarity to the other donor. Statistical significance between the groups are indicated with an asterisk (patient similarity to own donor vs. donor intra-individual similarity) and cross variation (patient similarity to own donor vs. patient similarity to other donors) is shown with standard error of mean (SEM). **b** Patient faecal samples present donor-specific microbial signatures in BaggedRDA analysis

One of the main aims of this work was to evaluate the long-term persistence of the transplanted microbiota. To address this, we calculated the Spearman correlations measuring microbial similarity between three groups, namely the similarity between the donated sample and its recipient, the donors within-subject similarity against the donated sample over time, and similarity between the patient and other donors of the study. The high similarity between patients’ and their donated sample throughout the follow-up period was striking (Fig. 3a). As early as 3-days after the treatment there was a 95.0 % similarity between the patients’ microbiota and the donated faeces, compared to the 81.8 % similarity pre-FMT (*P* < 0.05). Importantly, similarity to the other donors was significantly lower than to the own donors (*P* < 0*.*05) and, furthermore, this similarity was retained throughout the study period (Fig. 3a). In addition, the overall similarity between the patient–donor pairs (average 95.3 %) was found to be remarkably higher than what is generally observed for unrelated individuals determined using the same analysis pipeline (average 77.4 %, *P* < 0.05) [25, 28, 34–36]. Analysis of the microbiota stability at individual level showed that it had high resilience after FMT and, in three out four patients receiving antibiotics during the follow-up period, the microbiota was able to recover from the occasional antibiotic treatment for other indications (Additional file 1: Figure S4). As an exception, P13, who has Crohn’s disease and received three courses of antibiotics, both of which are known to affect microbiota, had reduced stability (Additional file 1: Figure S4) and was therefore excluded from the cohort stability analysis.

Due to the high similarity between the donors and their patients, we investigated the possibility of microbial signatures in the patients that would be specific to their own donor. Using baggedRDA, we found that the patients could be separated according to their donor and observed 24 genus-like taxa to cause this separation (Fig. 3b). For example, bacteria related to *Faecalibacterium prausnitzii*, *Ruminococcus lactaris* and *Collinsella* were increased in the patients from D3. These signatures remained throughout the follow-up period.

### Commonly acquired bacterial taxa

**Fig. 4:**
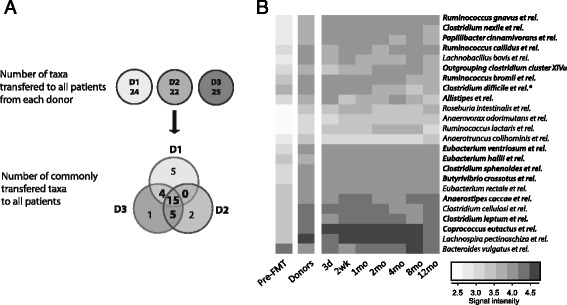
The commonly acquired bacteria after faecal microbiota transplantation (FMT). **a** Flowchart showing how the commonly acquired bacteria were identified. **b** Heatmap showing the bacterial taxa, abundance and the stability of the therapeutic core. The bacterial groups shown with bold text were increased in all patients and the others were increased in patients from two out of three donors. *The bacteria belonging to *C. difficile* group include eight commensal species and uncultured representatives (see Additional file 1), which produced the detected signal. *C. difficile* per se was absent from all donors and patients post-FMT

The universal donor approach of this study allowed identification of similarities introduced by the FMT. More specifically, we were able to identify genus-like bacterial taxa that were absent in the patients prior to treatment but introduced to the patients post-FMT (Fig. 4a). Each donor and their patients were first compared separately to achieve the donor-specific transplanted core microbiota. We then compared the three donor-specific cores (Fig. 4b) and found that 24 genus-like taxa from four phylum-like groups were absent in patients prior to FMT and introduced by the treatment to at least two out of the three donors (Fig. 4b). Fifteen of these taxa were present in all patients after treatment. The commonly acquired bacteria included some well-studied butyrate producers such as *Eubacterium hallii* and *Roseburia intestinalis.*

## Discussion

Our study addressed the microbiological mechanisms underlying the FMT treatment for rCDI. We showed, for the first time, that FMT has long-term effects on the microbiota and offers a means to modify it relatively permanently. The rapid changes induced by FMT explain the prompt and high clinical efficacy – it drastically altered the patients’ intestinal microbiota by restoring the anaerobic community. Patients’ faecal microbiota prior to FMT was dominated with facultative anaerobic bacteria such as Bacilli and Proteobacteria, which are known for their proinflammatory properties [37]. Post-FMT, their microbiota composition resembled that of the donors as early as 3 days following transplantation, containing bacteria typical for a healthy microbiota such as strict anaerobes from the Clostridium clusters IV and XIVa. These observed changes confirmed previous findings [14, 38] and, importantly, we were able to show that these modifications persisted long-term. We also addressed the effects induced by FMT on the rectal mucosa, which have not been studied previously. Furthermore, our universal donor approach allowed the identification of commonly acquired bacterial taxa, potentially underlying the treatment efficacy.

Antibiotics suppress anaerobic commensals and induce profound changes to microbiota, resulting in loss of colonisation resistance [39, 40]. We observed a similar effect in patient P3, who mistakenly took vancomycin after the first FMT. The transplanted microbiota was unable to engraft and there was no change in the microbial composition before the second FMT treatment. We also showed that the patients’ microbiota composition pre-FMT represents the effects of multiple antibiotic treatments, including low diversity and depletion of anaerobes. The FMT-treatment restored these levels very rapidly.

The novel mucosal microbiota findings showed that similar to the faecal microbiota, FMT restored the anaerobic bacterial community due to the increase of Bacteroidetes. The faecal and mucosal tissues are distinct communities and have specific microbial compositions [41, 42]. Therefore, it was not surprising that a sub-population of the transplanted microbiota was selected to the mucosal compartment. Further, our in vitro experiment showed that the epithelium-adherent fraction of faecal microbiota was enriched in Bacteroidetes. This group is abundant in the healthy intestinal mucosa and is known to enforce epithelial integrity [43] and maintains immunological homeostasis [44, 45]. Thus, it can be hypothesised that the increase of Bacteroidetes in the mucosa was part of the efficacy of the FMT treatment.

One of the main findings of this study was the high similarity in the microbiota profiles between patients and their own donors that lasted throughout the 1-year follow-up. This was not altered even by antimicrobial treatments taken by some patients during the follow-up period. The microbial stability was effected by the antibiotics, but it recovered to its original composition, in line with recent observations with healthy subjects [40]. Regardless of the antibiotics, we were able to identify specific donor-derived bacterial signatures, which persisted throughout the follow-up. This surprisingly high similarity between the donor-patient pair led us to speculate that there is no major selection pressure from the host to alter the transplanted microbial composition. The hypothesis could be that the transplant provides a functional microbial ecosystem, which outweighs the individual-based bacterial selection.

Previously, three FMT-trials have addressed the engraftment of donors’ microbiota in patients, with shorter 4- to 6-month follow-up periods and less detailed microbial analysis [17, 38]. Our comprehensive investigation extends the previous preliminary observations on the establishment of donors’ microbiota post-FMT; both the high patient–donor similarity and the donor-specific bacterial signatures in patients indicate a long-term establishment of the donors’ microbiota. This is in line with a recent metagenomics study that revealed colonisation of donor bacteria at strain level persisting for 3 months after FMT treatment [46]. Since one of the characteristics of a healthy microbiota is its resilience to change [35], it was unexpected that the donors’ microbiota was so strongly established and maintained. Our hypothesis is that the depletion of the microbiota with broad-spectrum antibiotics and bowel cleansing creates an open ecological niche for the transplanted microbiota. This novel finding on the long-term stability is promising when considering other indications where changing the intestinal microbiota composition could be used as a potential treatment.

One of our main aims was to determine a group of bacteria necessary for the resolution rCDI. This was addressed by the universal study setup, where faecal preparations from three donors were used to treat several patients, allowing better evaluation of the commonly acquired bacteria, which were transferred to all patients. We identified 24 bacterial taxa that were absent in patients before the treatment and present afterwards. Thus, it would be plausible to hypothesise that such a specific subpopulation within the complex faecal microbiota could underlie the treatment efficacy of FMT for rCDI. This commonly acquired core identified in our study was taxonomically diverse and included bacterial genera from four major phyla. The therapeutic core determined in our study showed considerable overlap with health-associated microbial cores determined in other studies [47], highlighting its potential in restoring health.

The impact of these 24 taxa to intestinal health potentially lies in their ecological functions and nutrient utilisation networks as well as immunomodulatory capacity. One of these genera, *Bacteroides* spp. has been previously found to increase significantly after FMT for rCDI and to have a key role in restoring the intestinal ecosystem [14]. Our findings on the increase of *Bacteroides* spp. in the mucosa also underline their importance in maintaining intestinal homeostasis. There is evidence that the human commensal *B. fragilis* fortifies epithelial integrity [43] and, more recently, the bacterium was shown to interact with intestinal mucosa to suppress inflammation [48]. Furthermore, mice studies have shown that the Bacteroidetes taxa are required in successful colonisation of a health-associated *Faecalibacterium prauznitzii* [49]*.*

The majority (22/24) of the commonly transplanted bacterial taxa belonged to three Clostridium clusters (Firmicutes). The Clostridium taxa of the therapeutic core have been shown to play key roles in the nutrient utilisation networks and, therefore, can be considered to be essential for the general restoration of the complex ecosystem [50–52]. For example, the therapeutic core bacteria *Eubacterium*, *Coprococcus*, *Anaerostipes* and *Ruminococcus* spp. are known to participate in bacterial cross-feeding pathways that are responsible for the production of short-chain fatty acids (SCFA) – the major microbial metabolites from carbohydrate fermentation [50]. Concurrently, with the appearance of the therapeutic core taxa, we also observed a more than 20-fold increase in *Ruminococcus obeum* and *Subdoligranulum variable*, both of which are major SCFA-producing bacteria in the gut [50]. SCFAs promote intestinal homeostasis by both strengthening epithelial cell layer integrity and stimulating regulatory T cells [53]. Recently, Atarashi et al. [54] treated inflammatory colitis in a mouse model with a combination of 17 clostridial strains, which affected SCFA and regulatory T cell levels.

In summary, the therapeutic core seems to consist of intestinal bacteria that are able to regenerate key interaction-networks within the microbiota and consequently restore the complex intestinal ecosystem that carries out essential functions for the host and provides colonisation resistance against pathogens, especially *C. difficile.* Therefore, isolation and characterisation of these commensal bacteria would be of high importance when developing microbiota-based therapies for rCDI. We consider that there are multiple alternatives to combine intestinal bacterial strains as an effective bacteriotherapy mixture.

## Conclusions

The microbiota changes both in faeces and mucosa explain the rapid clinical recovery of all patients and the superior long-term efficacy over previous antibiotic treatments. Our results indicate that a specific combination of bacterial taxa seems to underlie the treatment efficacy of FMT for rCDI. This is the first study to show that subject’s microbiota could be modified in the long-term to resemble that of the donor. Currently, FMT treatment is considered for several other indications than just the treatment of rCDI. Therefore, our findings give insights into the possibilities of reshaping patients’ microbiota relatively permanently.

## Additional file

Additional file 1:
**Methods** – Adherence of bacteria to intestinal epithelium, details of sequencing processing, phylogenetic assignment of species belonging to the Clostridium difficile genus level group). **Results** – results of P3 analysis. **Figure S1:** Detailed description of the patients A) recruitment flow chart B) list of collected samples. **Figure S2:** Microbial changes in the patient P3 during the trial A) microbial composition B) PCA, C) microbial diversity. **Figure S3:** The mucosal bacteria separating the patients pre- and post-FMT biopsies determined with BaggedRDA. **Figure S4:** Patients individual microbial stability. **Table S1:** Detailed patient demographics. **Table S2:** Significant differences between the pre- and post-FMT samples on genus-like taxonomical level. **Table S3:** Microbial diversity of the mucosal samples. (PDF 2230 kb)

## Abbreviations

BaggedRDA: Bootstrap aggregated redundancy analysis
FMT: Faecal microbiota transplant
PCA: Principle component analysis
rCDI: Recurrent *Clostridium difficile* infection
SCFA: Short-chained fatty acids
