# Supplementary material for: Long-term effects on luminal and mucosal microbiota and commonly acquired taxa in faecal microbiota transplantation for recurrent Clostridium difficile infection
Source: BMC Med. 2016 Oct 11;14:155. doi: 10.1186/s12916-016-0698-z (PMC5057499; doi:10.1186/s12916-016-0698-z)
Supplement: Additional file 1: — Methods – Adherence of bacteria to intestinal epithelium, details of sequencing processing, phylogenetic assignment of species belonging to the Clostridium difficile genus level group). Results – results of P3 analysis. Figure S1: Detailed description of the patients A) recruitment flow chart B) list of collected samples. Figure S2: Microbial changes in the patient P3 during the trial A) microbial composition B) PCA, C) microbial diversity. Figure S3: The mucosal bacteria separating the patients pre- and post-FMT biopsies determined with BaggedRDA. Figure S4: Patients individual microbial stability. Table S1: Detailed patient demographics. Table S2: Significant differences between the pre- and post-FMT samples on genus-like taxonomical level. Table S3: Microbial diversity of the mucosal samples. (PDF 2230 kb) [file 12916_2016_698_MOESM1_ESM.pdf]

Additional file 1 for the publication:

"Long-term effects on luminal and mucosal microbiota and commonly acquired taxa in fecal microbiota transplantation for recurrent Clostridium difficile infection "

Methods

Adherence of donor fecal bacteria to intestinal epithelium *in vitro*

Cultivation of Caco-2 cells and *in vitro* bacterial adhesion experiment:

Bacterial material from the donor 3 fecal sample were allowed to bind to human enterocyte cell line Caco-2 by adding a volume of 200 µl of fecal suspension on 7-day-old Caco-2 cell culture and incubating these for one hour at 37°C in anaerobic cabinet. For the adhesion experiment, Caco-2 cells had been cultivated on 96-well tissue culture plates with RPMI-1640 culture medium supplemented with 2 mM L-glutamine, 20 % heat inactivated fetal calf serum, 100 U/ml penicillin-streptomycin, 1 % nonessential amino acids and 5 mM HEPES buffer, as described previously.[1] Prior to the adhesion experiment, the cell culture was first washed three times with culture medium without the supplements, and after the adhesion non-adherent bacteria were removed by washing the wells three times with PBS.

DNA sequencing and data processing from raw reads to taxonomically assigned OTUs:

Adhered bacteria were collected and DNA was extracted like previously described[2], followed by amplifying the region V1-V3 of 16S rRNA gene with the primers pA 5'-AGAGTTTGATCMTGGCTCAG-3' for the forward primer[3] and pD 5'-GTATTACCGCGGCTGCTG-3' for the reverse primer[4]. We used the Illumina MiSeq platform for sequencing. The raw reads were preprocessed with in-house scripts involving merging of forward and reverse reads to get paired-end sequences of over 435 bp. OTU-clustering and chimera filtering of paired-end sequences was done by UPARSE OTU-clustering software[5] using QIIME bioinformatics pipeline[6]. This resulted into 94200 sequences from the fecal sample and 78400 sequences from the adhered sample. In taxonomic assignment of OTUs, the newly published HITdb was used as a reference database.[7] We identified 374 and 303 unique OTUs from the fecal and adhered sample respectively.

Species belonging to the *Clostridium difficile* genus level group:

The probes assigned to the genus-like group entitled *Clostridium difficile* et al., contains, in addition to *C. difficile*, eight commensals*; Clostridium bartlettii, Clostridium bifermentans, Clostridium difficile, Clostridium ghoni, Clostridium glycolicum, Clostridium hiranonis, Clostridium irregularis, Clostridium sordellii* and *Eubacterium tenue* in addition to seven uncultured representatives. Majority of the microarray signal of the donor and post-FMT samples came from these uncultured representatives, *Clostridium bartlettii* and *Clostridium hiranonis*. *C. difficile per se* was absent from all donors and patients post-FMT.

Results

Patient P3:

Patient P3 mistakenly consumed vancomycin (vancomycin 125mg four times a day for 14 days). This resulted into a relapse after a week the antibiotics were discontinued. As a treatment she received a second FMT from the same donor, which resolved the rCDI and remained asymptomatic throughout the trial. The vancomycin treatment enabled the transplanted microbiota to function appropriately and the microbial composition of this patient did not start to resemble that of the donors after the FMT while consuming vancomycin (additional figure 2B). The microbiota composition remained distorted and over taken by Bacilli and Proteobacteria and was underrepresented with facultative anaerobes such as the Clostridium clusters IV and VIXa. Moreover, microbial diversity remained very until a success full FMT (additional figure 2C).

Additional Figure 2: Microbial changes in the patient P3 during the trial. A) Microbial composition in fecal samples. Patient took 14d course of vancomycin after the initial FMT. B) PCA from genus level bacterial groups in fecal samples, pre-FMT samples that were influenced by the vancomycin treatment colored green and post-FMT samples colored blue, sample coding (1-12) same as in panel A C) Microbial diversity from in fecal samples

References:

1. Kainulainen V, Reunanen J, Hiippala K, Guglielmetti S, Vesterlund S, Palva A, Satokari R: **BopA does not have a major role in the adhesion of Bifidobacterium bifidum to intestinal epithelial cells, extracellular matrix proteins, and mucus**. *Appl Environ Microbiol* 2013, **79**(22):6989-6997.
2. Salonen A, Nikkilä J, Jalanka-Tuovinen J, Immonen O, Rajilic-Stojanovic M, Kekkonen RA, Palva A, de Vos WM: **Comparative analysis of fecal DNA extraction methods with phylogenetic microarray: effective recovery of bacterial and archaeal DNA using mechanical cell lysis**. *J Microbiol Methods* 2010, **81**(2):127-134.
3. Edwards U, Rogall T, Blocker H, Emde M, Bottger EC: **Isolation and direct complete nucleotide determination of entire genes. Characterization of a gene coding for 16S ribosomal RNA**. *Nucleic Acids Res* 1989, **17**(19):7843-7853.
4. Lane DJ, Pace B, Olsen GJ, Stahl DA, Sogin ML, Pace NR: **Rapid determination of 16S ribosomal RNA sequences for phylogenetic analyses**. *Proc Natl Acad Sci U S A* 1985, **82**(20):6955-6959.
5. Edgar RC: **UPARSE: highly accurate OTU sequences from microbial amplicon reads**. *Nat Methods* 2013, **10**(10):996-998.
6. Caporaso JG, Kuczynski J, Stombaugh J, Bittinger K, Bushman FD, Costello EK, Fierer N, Pena AG, Goodrich JK, Gordon JI *et al*: **QIIME allows analysis of high-throughput community sequencing data**. *Nat Methods* 2010, **7**(5):335-336.
7. Ritari J, Salojarvi J, Lahti L, de Vos WM: **Improved taxonomic assignment of human intestinal 16S rRNA sequences by a dedicated reference database**. *BMC Genomics* 2015, **16**(1):1056.

A

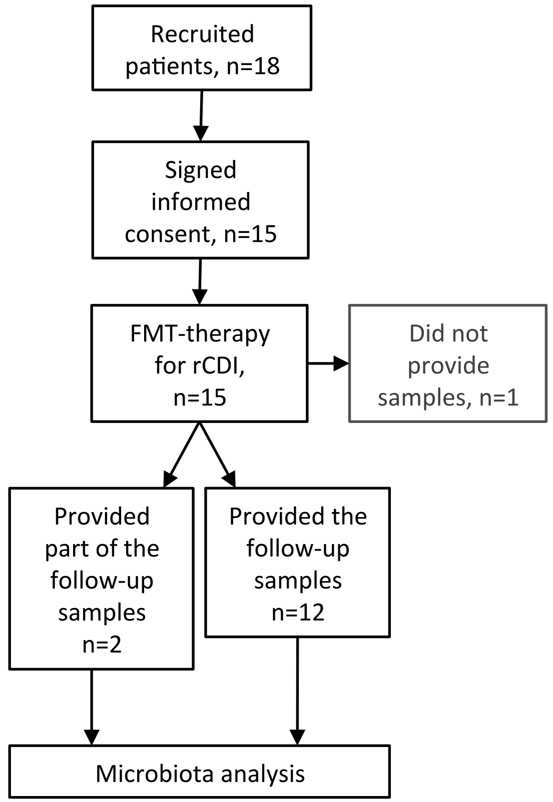

B

	0	3d	2wk	1mo	2mo	4mo	8mo	12mo
P1	X	x	x	X	x	x	-	-
P2	X	x	x	X	x	x	x	x
P3-1st	X	x	x	x	-	-	-	-
P3-2nd	X	x	x	x	x	x	x	x
P4	X	x	x	X	-	-	-	-
P5	X	x	x	X	x	x	x	x
P6	X	x	x	X	x	x	x	x
P7	X	-	x	X	x	x	x	x
P8	X	x	x	X	x	x	x	x
P9	X	x	x	x	x	x	x	x
P10	X	x	x	X	x	x	x	x
P11	X	-	x	x	x	x	x	x
P12	X	x	x	X	x	x	x	x
P13	-	x	x	X	x	x	x	x
P14	-	x	x	X	x	x	x	x
D1	x	x	x	x	x	-	x	x
D2	x	x	x	x	x	x	x	x
D3	x	x	x	x	x	x	x	x

Additional Figure 1: Description of the participants A) Recruitment flowchart B) The collected fecal samples are marked with a cross and the collected rectal biopsy samples marked with large cross. Patient P3 had two FMTs and 4 samples from the 1t FMT are marked with P3-1st and the sample from the second FMT are marked P3-2nd . See the main text for details.

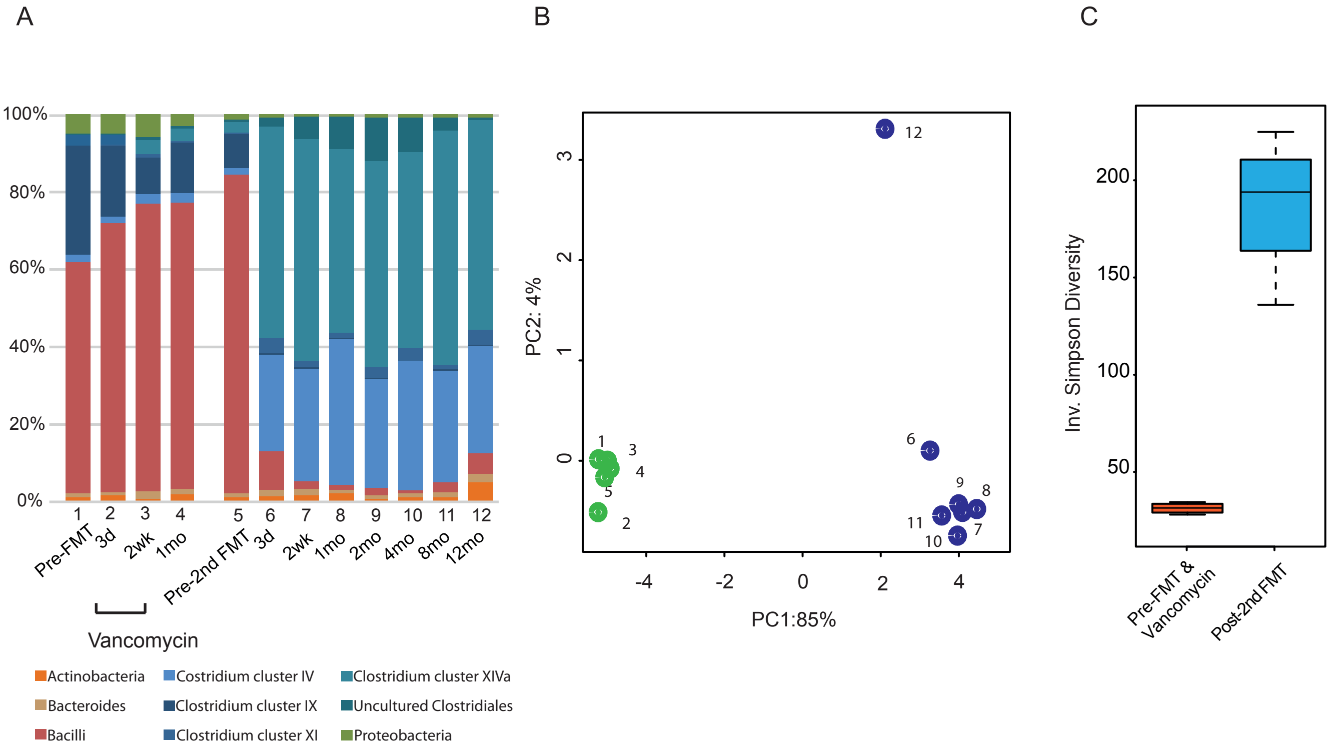

Additional file 1: Figure S2: Microbial changes in patient P3 during the trial. A) Microbial composition in fecal samples. Patient took 14d course of vancomycin after after the initial FMT. B) PCA from genus level bacterial groups in fecal samples, pre-FMT samples that were influenced by the vancomycin treatment coloured green and post-FMT samples coloured blue, sample coding (1-12) same as in panel A C) Microbial diversity from in fecal samples before succesful FMT (samples 1-5) and after 2nd FMT (samples 6-12)

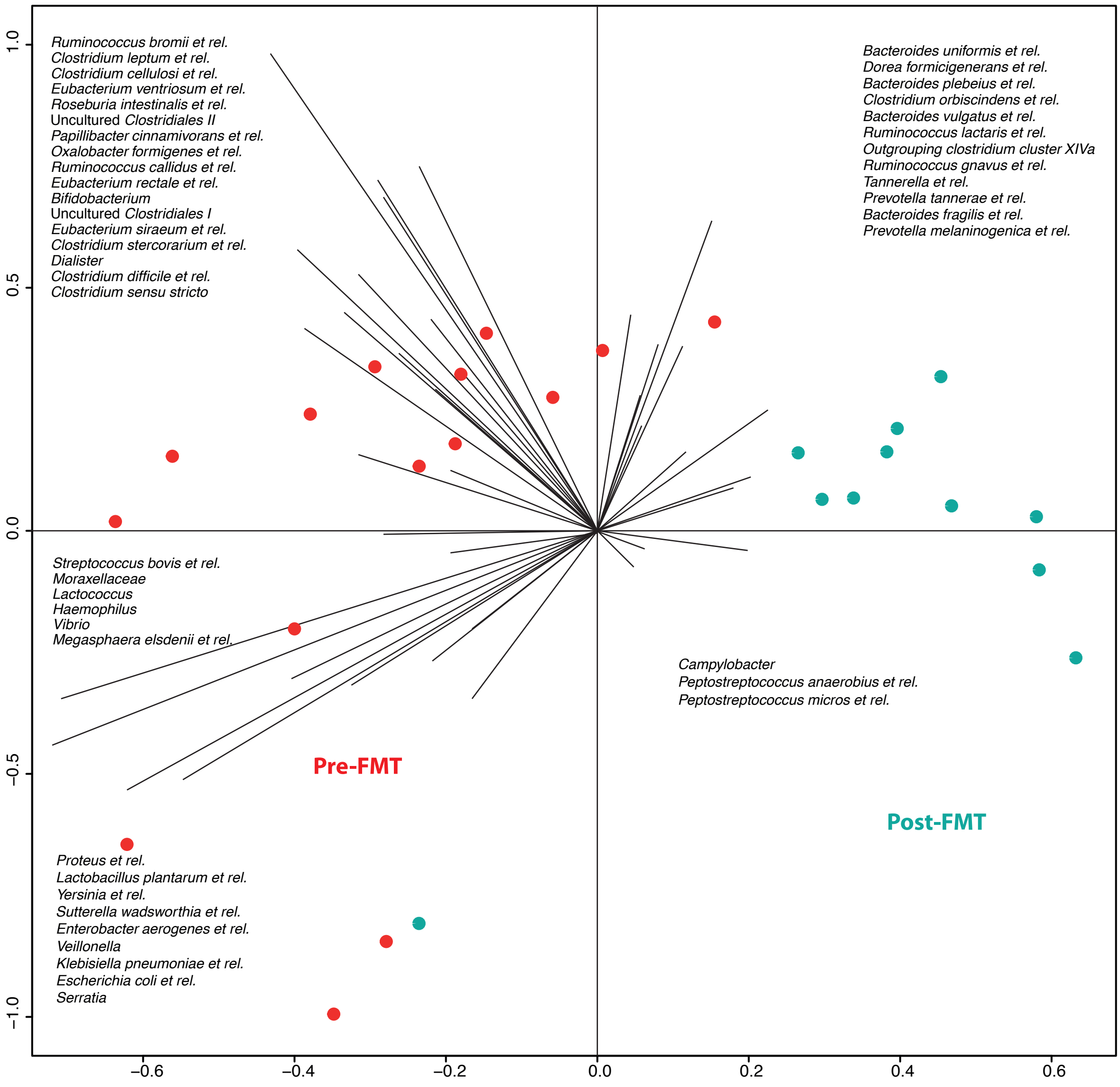

Additional file 1: Figure S3: The group of mucosal bacteria separating the patients pre- and post-FMT biopsies determined with BaggedRDA

Additional file 1: Figure S4

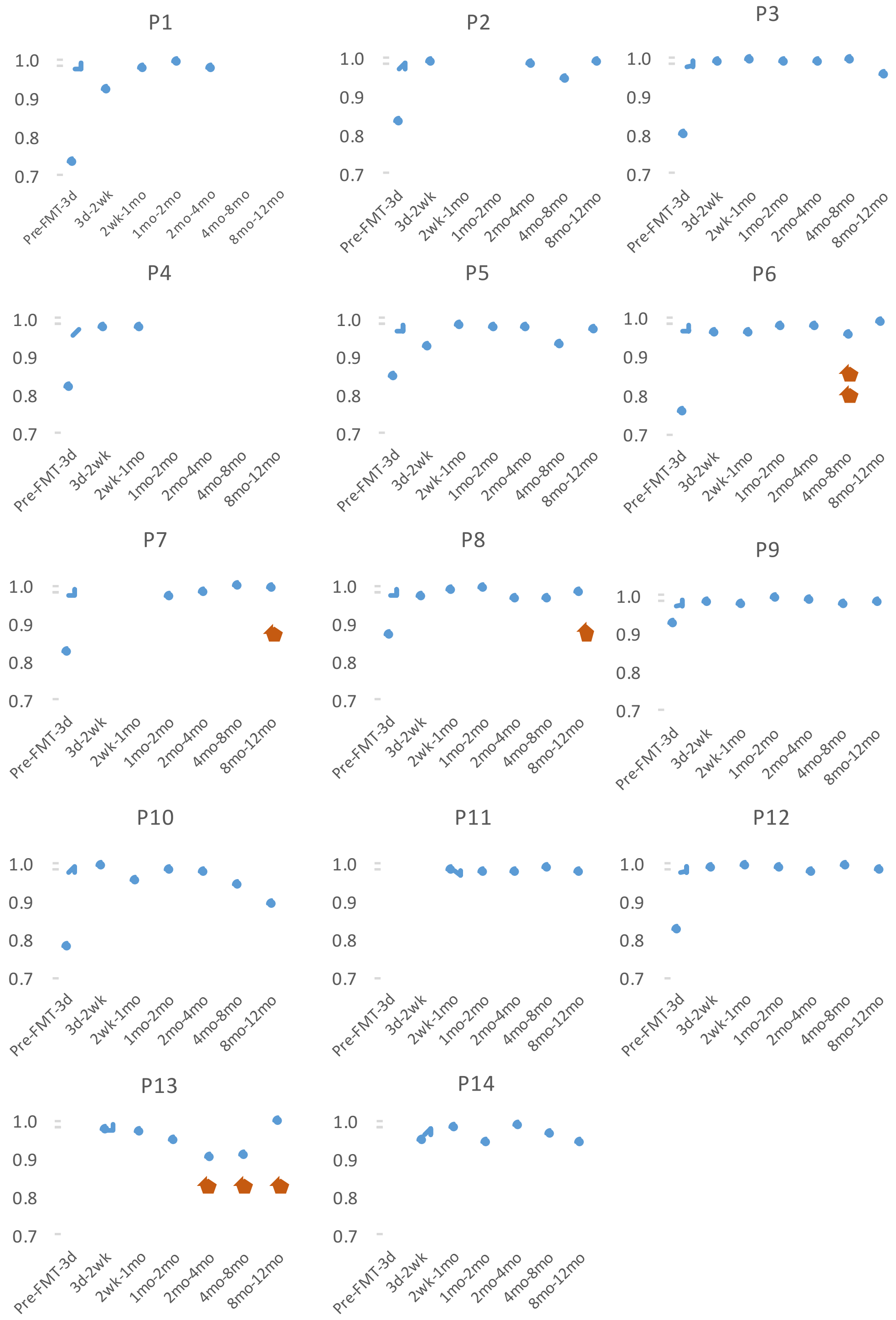

Supplementary figure 4: Stability of intestinal microbiota after FMT as determined with spearman correlation between consecutive time points. Individuals antibiotic treatments are indicated with red bullets (see suppmentary table 1 for further information on patient characteristics and specific antibiotic treatments).

Supplementary tables for the publication: " *Long-term effects on luminal and mucosal microbiota and commonly acquired taxa in fecal microbiota transplantation for recurrent Clostridium difficile infection* "

Additional file 1: Table S1: Detailed patient demographics

Patient	Age, y	Sex	days from frist CDI to FMT	Nr. of relapses	Ribotype	Antibiotics during the follow-up sampling	Index antibiotics	Other
P1	63	Male	175	4	027 negative	no	No data	-
P2	45	Male	157	5	027 negative	no	No data	Ulcerative colitis, in remission during the follow-up
P3	88	Female	133	4	027 negative	yes, vancomycin after 1st FMT	levofloxacin	Chronic renal insufficiency
P4	82	Female	269	5	027 negative	no	No antimicrobial agent	-
P5	81	Female	312	4	027 positive	no	No data	-
P6	58	Female	124	4	027 negative	yes, nitrofurantoin 2x75 mg/day for urinary tract infection, taken twice at 6, 7 mo post-FMT	clindamycin	-
P7	67	Male	146	6	027 negative	yes, ceftriaxone + meropenem+doxycycline for pneumoniae, taken 9 mo post-FMT	clindamycin+levofloxacin	Chronic renal insufficiency
P8	31	Female	83	3	027 negative	yes, nitrofurantoin 2x75 mg/day for urinary tract infection, taken 12 mo post-FMT	amoxicillin + sephalexin	Ulcerative colitis, in remission during the follow-up
P9	35	Female	67	2	027 negative	no	No data (travellers diarrhoea)	Crohns´disease, in remission during the follow-up
P10	81	Male	137	5	027 negative	no	No antimicrobial agent	Chronic renal insufficiency
P11	80	Female	119	3	027 negative	no	penicillin V	-
P12	20	Female	124	5	027 negative	no	clindamycin	-
P13	57	Female	42	1	027 negative	yes, nitrofurantoin 2x75 mg/day for urinary tract infection, taken three times 4, 9 and 11 mo post-FMT	doxycycline	Crohns´ disease, in remission during the follow-up
P14	44	Female	134	3	027 negative	no	sephalexin	-

Additional file 1: table S2: Significant differenes between the pre- and post-FMT samples

			Post-FMT							
**Phylum like taxa**	**Genus like taxa**	**Pre-FMT**	**3d**	**2wk**	**1mo**	**2mo**	**4mo**	**8mo**	**12mo**	**Effect of FMT**(Average of post-FMT/Pre-FMT)
Actinobacteria	*Bifidobacterium*	2781	18824	8371	22255	22372	19776	10182	5507	5.51
	Collinsella	246	743	758	1150	1595	1867	1297	725	4.72
	Eggerthella lenta et rel.	260	579	546	968	1197	1023	784	631	3.14
Bacilli	*Enterococcus*	1074	395	377	538	354	381	336	304	-2.88
	Granulicatella	353	120	97	112	67	90	58	60	-4.42
	Lactobacillus gasseri et rel.	1926	806	732	725	743	615	573	539	-2.91
	Lactobacillus plantarum et rel.	35008	1784	1304	1542	1370	1116	1379	1965	-24.18
	Lactobacillus salivarius et rel.	286	232	209	236	147	149	119	124	-1.77
	Lactococcus	347	144	141	248	237	165	140	138	-2.12
	Streptococcus bovis et rel.	36623	16357	12640	8018	7738	8985	9684	8307	-3.81
	Streptococcus intermedius et rel.	829	585	439	497	457	528	406	436	-1.76
	Streptococcus mitis et rel.	11581	7050	4233	3248	3665	3872	3741	3636	-2.91
	Weissella et rel.	211	153	162	170	167	175	148	141	-1.33
Bacteroidetes	*Allistipes et rel.*	869	3516	1792	3414	2713	2973	3043	1655	3.14
	Bacteroides fragilis et rel.	657	1945	984	2035	2195	1653	1806	1262	2.58
	Bacteroides intestinalis et rel.	57	260	116	186	160	154	162	118	2.90
	Bacteroides ovatus et rel.	533	1678	881	1628	1608	1412	1787	1048	2.69
	Bacteroides plebeius et rel.	313	1256	691	1287	1152	1153	1425	645	3.47
	Bacteroides splachnicus et rel.	688	1360	941	1327	1211	1339	1408	946	1.77
	Bacteroides stercoris et rel.	272	688	418	661	700	631	595	415	2.16
	Bacteroides uniformis et rel.	146	1580	573	1138	1075	861	1069	637	6.77
	Bacteroides vulgatus et rel.	1157	8539	4042	8820	8477	7133	13006	5897	6.91
	Tannerella et rel.	421	836	601	1092	1051	1043	1147	654	2.18
Clostridium cluster III	*Clostridium stercorarium et rel.*	220	1016	1276	1574	1796	1721	1407	924	6.30
Clostridium cluster IV	*Anaerotruncus colihominis et rel.*	524	853	1023	1372	900	1247	1146	1319	2.14
	Clostridium cellulosi et rel.	1188	12016	13036	13922	12246	8640	7262	12033	9.52
	Clostridium leptum et rel.	943	7863	10656	17090	13486	10555	8036	11895	12.05
	Clostridium orbiscindens et rel.	1480	16559	19744	28807	24588	18815	23225	27923	15.41
	Faecalibacterium prausnitzii et rel.	2649	17875	24533	38693	26842	18575	49922	13776	10.26
	Oscillospira guillermondii et rel.	1879	12498	14886	26181	14150	12826	16571	20541	8.95
	Papillibacter cinnamivorans et rel.	552	7141	6828	6692	6113	4323	5621	5521	10.93
	Ruminococcus bromii et rel.	298	4854	6674	5929	3246	3013	2000	2486	13.51
	Ruminococcus callidus et rel.	784	3667	5014	5445	4058	5873	8270	4371	6.69
	Sporobacter termitidis et rel.	1440	7310	10627	18400	9597	10783	8726	14986	7.98
	Subdoligranulum variable at rel	1754	39677	27473	40956	26203	34686	54858	48812	22.21
Clostridium cluster IX	*Dialister*	2104	428	312	386	451	423	461	515	-5.06
	Megasphaera elsdenii et rel.	557	284	235	277	269	268	249	244	-2.15
	Veillonella	12635	566	220	380	290	298	304	287	-40.64
Clostridium cluster XI	*Anaerovorax odorimutans et rel.*	376	1404	1692	2067	1860	2037	1582	1914	4.77
Clostridium cluster XIVa	*Anaerostipes caccae et rel.*	840	7573	10922	13568	12615	10381	11769	10144	13.08
	Bryantella formatexigens et rel.	1547	6712	8893	9333	7761	7797	12890	5008	5.39
	Butyrivibrio crossotus et rel.	986	5912	5646	8653	9153	7760	9637	6489	7.72
	Clostridium nexile et rel.	478	4907	5240	7885	7207	9708	6207	4086	13.52
	Clostridium sphenoides et rel.	1115	8357	8611	10403	9447	9872	10191	6855	8.16
	Clostridium symbiosum et rel.	2001	9964	15092	16492	15151	14799	17889	18957	7.73
	Coprococcus eutactus et rel.	1010	31734	29189	21761	20123	29303	30199	12470	24.71
	Dorea formicigenerans et rel.	1821	16083	21698	20967	21267	23234	21967	18116	11.25
	Eubacterium hallii et rel.	447	5808	8935	9756	9525	8916	6570	6944	18.02
	Eubacterium rectale et rel.	956	4098	7094	6831	6933	5871	7744	4024	6.37
	Eubacterium ventriosum et rel.	446	4074	7070	9855	8244	7379	5676	7767	16.02
	Lachnobacillus bovis et rel.	631	2570	3581	4353	3207	3637	4100	3436	5.63
	Lachnospira pectinoschiza et rel.	1164	11190	18560	25247	21533	21269	25547	10234	16.39
	Outgrouping clostridium cluster XIVa	823	3057	4406	4679	5262	5611	5733	4805	5.82
	Roseburia intestinalis et rel.	271	1674	2372	3401	2047	2448	2147	1665	8.32
	Ruminococcus gnavus et rel.	517	6304	5161	8530	7732	8442	6602	6183	13.53
	Ruminococcus lactaris et rel.	194	1164	1679	1862	2117	1965	1849	1040	8.58
	Ruminococcus obeum et rel.	3320	84061	87782	73617	71705	75280	88024	55168	23.05
Clostridium cluster XVIII	*Clostridium ramosum et rel.*	219	340	278	566	572	496	336	337	1.91
	Coprobacillus catenaformis et rel.	205	532	463	1119	1220	1082	567	331	3.70
Fusobacteria	*Fusobacteria*	572	350	343	431	396	385	357	347	-1.54
Proteobacteria	*Enterobacter aerogenes et rel.*	3849	868	490	987	1052	797	829	521	-5.24
	Escherichia coli et rel.	2101	747	517	898	797	673	706	539	-3.12
	Klebisiella pneumoniae et rel.	1964	547	283	588	601	416	443	320	-4.63
	Oxalobacter formigenes et rel.	296	610	594	1017	851	803	598	522	2.41
	Proteus et rel.	451	316	308	365	358	345	329	300	-1.37
	Serratia	204	76	42	84	73	54	70	42	-3.46
	Yersinia et rel.	282	190	160	240	244	190	186	164	-1.47
Uncultured Clostridiales	Uncultured *Clostridiales I*	1267	3003	5109	8241	3307	3185	2138	2103	3.05
	Uncultured *Clostridiales II*	1338	4070	5331	6668	4162	4578	4260	5616	3.70

Additional file 1: Table S3: Shannon diversity from the rectal mucosal samples.

	Shannon diversity	
Patient	Pre-FMT	Post-FMT
P1	253	218
P2	250	178
P4	160	119
P5	256	221
P6	257	230
P7	94	196
P8	244	244
P10	239	174
P12	250	167
P13	224	141
P14	72	206
